# Outcomes of Early-stage Glottic Carcinoma Treated with Radiation Therapy: A Single Institution Experience

**DOI:** 10.7759/cureus.3444

**Published:** 2018-10-12

**Authors:** Mubarak AlQahtani, Ahmed M. Maklad, Muhammad Shuja, Khalid H AlQahtani, Hussain AlHussain, Saleh F AlDhahri, Abdullah AlAmro, Moamen M Aly, Mutahir A Tunio, Amal Marie, Feras Alkholaiwi, Nasser Alobida, Ayman A Elghazaly, Yasser Bayoumi

**Affiliations:** 1 Department of Head and Neck Surgery, King Saud University, Riyadh, SAU; 2 Department of Radiation Oncology, King Fahad Medical City, Riyadh, SAU; 3 Department of Head and Neck Surgery, Al Imam Mohammad Ibn Saud Islamic University, Riyadh, SAU; 4 Department of Head and Neck Surgery, King Fahad Medical City, Riyadh, SAU; 5 Miscellaneous, Alfaisal University, Riyadh, SAU

**Keywords:** early glottic carcinoma, radiotherapy, treatment outcomes

## Abstract

Objective: To evaluate the outcomes of radical intent radiation therapy in early glottic carcinoma (EGC), including local control rate (LCR), disease-free survival (DFS), death specific free survival (DSFS), and overall survival (OS) rates, in Saudi patients treated at a single institution.

Materials and methods: This is an institutional review board (IRB) approved, retrospective study of 27 patients with T1-2 N0 M0, early glottic carcinoma (EGC) who were treated from 2010 to 2015 at our institution with different radiotherapy (RT) fractionation regimens. The regimens included six different fractionation schedules of radiotherapy (RT): 50 Gy (20 x 2.5 Gy) dose prescribed to 95% isodose line, 52.4 Gy (20 x 2.52 Gy), 63 Gy (28 x 2.25 Gy), 66 Gy (33 x 2 Gy), and 70 Gy (35 x 2 Gy). The cohort was stratified into two groups, ≤ 52.5 Gy (n=15) and > 52.5 Gy (n=12). The median follow-up of all patients was 31.7 months (range 7-82).

Results: The mean age of the cohort was 64.5 years (median 65, range: 41-83). Eleven patients (40.7%) had a history of smoking. The majority of the cohort was with T1a EGC (70.4%, n=19), and anterior commissure invasion was seen in three patients (11.1%). The mean RT doses were 55.6 Gy (range: 50-70). The five-year LCR, DFS, DSFS, and OS rates were 83.1%, 80.0%, 96.2%, and 92.6%, respectively. The LCR rates for those receiving a dose of 52.5 Gy or less were 61.3 months compared to 89.5 months for those who received more than 52.5 Gy (p=0.994). Non-smokers and patients with an unknown smoking history achieved a five-year LCR of 100%, while patients with a positive smoking history achieved a five-year LCR of 60.6% (p=0.044).

Conclusion: Radiation therapy for EGC in our patients showed reasonable five-year LCR with larynx preservation at 83.1%, DFS 80.0%, five-year OS rate 92.6%, and DSFS rate 96.2%. We found that smoking had a significant correlation with LCR. However, large prospective trials are warranted to evaluate the efficacy of overall treatment time, dose per fraction of above 2 Gy, and smoking effect.

## Introduction

Compared to the rest of the world, the incidence of laryngeal squamous cell carcinoma is much lower in the Kingdom of Saudi Arabia, likely due to the recent adoption of smoking habits in the population over the last three decades [1]. In one of the retrospective reviews of head and neck (H&N) malignancies conducted between 1987 and 2000 at King Fahad Specialist Hospital (KFSH) and Prince Faisal Oncology Center (PFOC), Buraidah, Saudi Arabia, it is reported that nine out of 135 (6.7%) H&N cancer patients were diagnosed with laryngeal carcinoma [2]. According to the Saudi Cancer Registry report 2012, laryngeal carcinoma constituted 1% of all site malignancies [3]. Among those, the majority of patients presented at advanced stages.

The true vocal cords (TVC) or glottic larynx is the most commonly involved subsite, depicting approximately two-thirds of laryngeal carcinoma [4]. Glottic carcinoma typically presents early, and unlike many other H&N malignancies, lack of lymphatic drainage in the glottis mucosa fetches small risk of lymph node involvement [5]. Early glottic carcinoma (EGC) is defined as tumor confined to one TVC (T1a), both vocal cords (T1b), or tumor that extends to supraglottic or subglottic with minimal functional impairment of TVC (T2) [6]. Radical radiation therapy is the appropriate initial modality for T1 and T2 lesions, with surgery reserved for salvage after failure.

Hypofractionated regimens are undertaken routinely for treating EGC [5,6]. One of the randomized trials demonstrated the superiority of modest hypofractionation with 2.25 Gy per fraction and, in large retrospective series, fraction sizes of ≥ 2.25 Gy compared favorably with other reported series [7-9]. Many United Kingdom (UK) series have reported high rates of local control with shorter hypofractionated schedules ranging from 50-52.5 Gy delivered in 16 fractions over three weeks for T1 lesions and 55 Gy in 20 fractions for T1 and T2 lesions [10-12]. On the other hand, hyperfractionated schedules have not shown significant improvement compared with conventional fractionation [13,14].

In our study, our aim was to evaluate the clinicopathological characteristics, the outcomes of radical radiation therapy including local control rate (LCR), disease-free survival (DFS), death specific free survival (DSFS), and overall survival (OS), rates in EGC in Saudi Arabian patients.

## Materials and methods

After a formal acceptance from the hospital ethical review committee, medical charts of patients with histopathologically confirmed early glottic cancer (T1 and T2) were reviewed retrospectively. Only those patients who received radiation therapy as their primary treatment were included in the study. Patients were treated with six different fractionation schedules of radiotherapy (RT): 50 Gy (20 x 2.5 Gy) dose prescribed to 95% isodose line, 52.4 Gy (20 x 2.52 Gy), 63 Gy (28 x 2.25 Gy), 66 Gy (33 x 2 Gy), and 70 Gy (35 x 2 Gy).

Data regarding demography, symptomatology, age, gender, smoking status, T-stage, histopathology, radiation therapy techniques and dose, follow-up duration, and treatment outcomes including local control rate (LCR), disease-free survival (DFS), death specific free survival (DSFS), and overall survival (OS) rates in early glottic carcinoma (EGC) were collected.

Statistical analysis

The primary endpoint was LCR. Secondary endpoints were DFS, DSFS, and OS. LCR was defined as the time from diagnosis to locoregional failure, DFS was defined as the time from diagnosis to locoregional failure, distant failure or death resulting from any disease, whichever occurred first, DSFS was defined as the time from diagnosis to death resulting from glottic disease, while OS was the time from diagnosis to death resulting from any cause or lost to follow-up, whichever occurred first. The survival curves were calculated using the Kaplan-Meier method, and the difference in survival curves was compared by using the log-rank test. Different categorical variables were compared with the chi-square (χ2) test. The level of significance was set at p < 0.05.

## Results

A total of 27 patients with early glottic cancers were treated at our institute from December 2010 to October 2015. The mean age of our cohort was 64.5 years (range 41-83). Almost half of our patients were aged 65 years and above (52%, n=14) with males contributing to nearly 89% of the cohort (n=24). Eleven patients (40.7%) had a positive smoking history.

We found hoarseness of voice as the most common presenting symptom in all the cases (100%). The majority of the cohort was with T1a EGC (70.4%, n=19) and with moderately differentiated squamous cell carcinoma histology (77.8%, n=21). Anterior commissure invasion was seen in three patients (11.1%). None of the patients had any nodal involvement or any metastatic disease.

All the patients were treated with radical radiation therapy as the first line of treatment. The mean radiation therapy doses were 55.6 Gy (range: 50-70). About 56% of patients (n=15) received more than 52.5 Gy as a total dose. The predominant radiation technique used was three-dimensional conformal radiation therapy (3D-CRT) in almost 70% (n=19) of the cases. The characteristics of the patients are given in the following table (Table 1).

**Table 1:** Patient characteristics N = number; TNM = tumor, node, metastasis; SCC = squamous cell carcinoma; Gy = Gray; RT = radiation therapy; 3D-CRT = 3 dimensional conformal radiation therapy

Variables	Names	N (%)
Age groups	Below 65 years	13 (48.14)
	More than 65 years	14 (51.85)
Gender	Male	24 (88.9)
	Female	3 (11.1)
Smoking status	Yes	11 (40.7)
	No	7 (25.9)
	Not known	9 (33.3)
TNM staging	T1a	19 (70.4)
	T1b	3 (11.1)
	T2	5 (18.5)
Histology	G2 SCC	21 (77.8)
	G3 SCC	6 (22.2)
Radiation therapy doses	= or < 52.5 Gy	15 (55.6)
	> 52.5 Gy	12 (44.4)
RT Technique	3D-CRT	19 (70.4)
	Volumetric Modulated Arc Therapy (VMAT)	8 (29.6)

Toxicity profile

Our cohort tolerated radiation therapy very well with none of them prolonging or interrupting their course than the conventional five days per week fractionation schedule. Acute radiation-induced grade 3 dermatitis was seen in one (3.7%), and grade 4 was seen in one patient (3.7%). Grade 3 acute mucositis was seen in three patients (11.1%), while grade 2 mucositis was observed in five patients (18.5%).

Local control rate (LCR)

**Figure 1:**
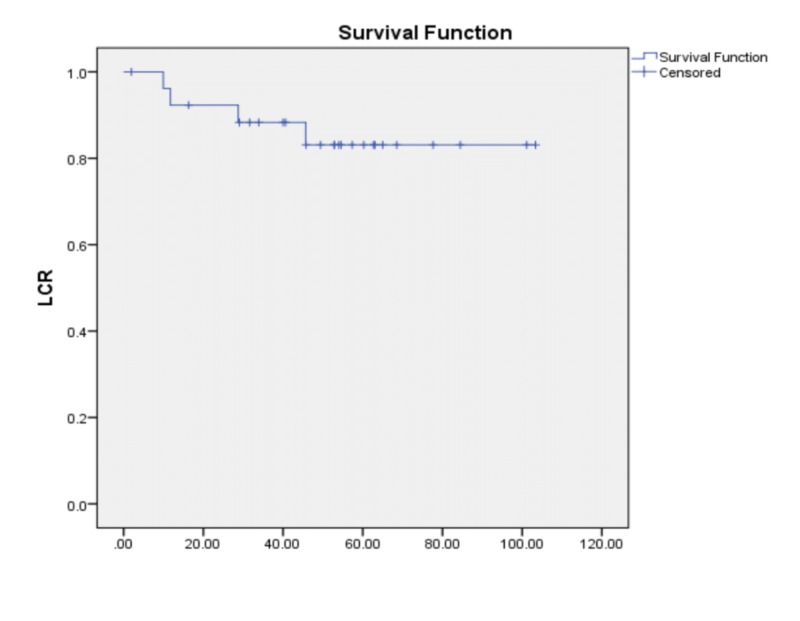
Overall local control rate LCR = Local control rate

The median duration of the follow-up was 52.83 months (range 2-103). A total of four patients (14.8%) had local recurrences with a median time to local recurrence 22.2 months from the completion of radiotherapy (range: 10-33). Two local recurrences were managed with salvage laryngectomy, while one patient refused surgery and another died of another primary (hepatocellular carcinoma). The five-year LCR of our cohort was 83.1% (Figure 1).

According to age, the mean LCR was 96.3 months for those below 65 years and 81.4 months for those 65 years or older. There was no statistically significant difference between these two groups (p= 0.25) and that could be explained by a lower number of patients. However, the curves showed some variation. Histopathology did not have any statistically conclusive effect on mean LCR, with 64.1 months for well-differentiated and 91.2 months for moderately differentiated cancers with a p-value of 0.761.

**Figure 2:**
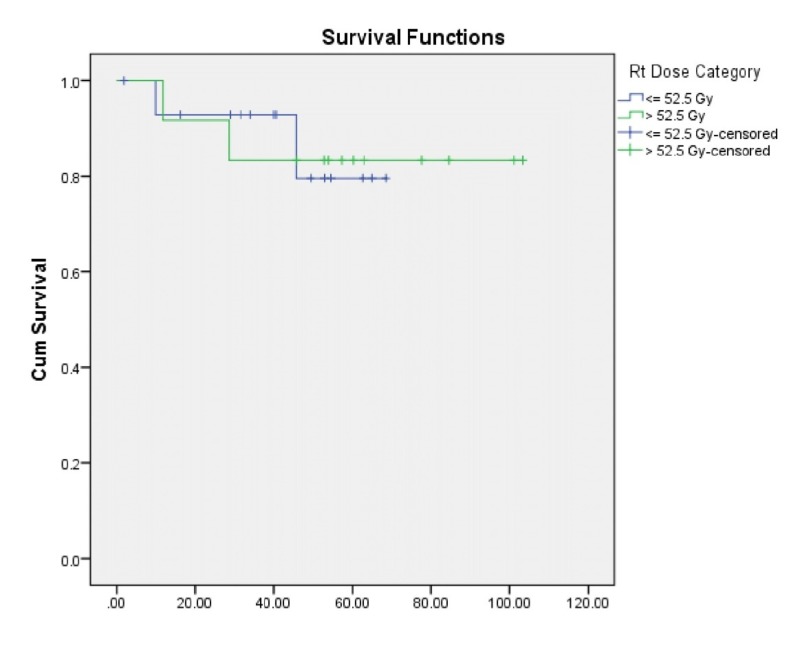
Local control rate based on radiation therapy doses

LCR for those receiving a dose of 52.5 Gy or less was 61.3 months compared to 89.5 months for those who received more than 52.5 Gy (p=0.994) (Figure 2). Regarding T stage, we noticed that all patients with T1b did well with a five-year LCR of 100%, while patients with T1a had a five-year LCR of 80.8% and T2 a five-year LCR of 80.0% with p=0.759.

**Figure 3:**
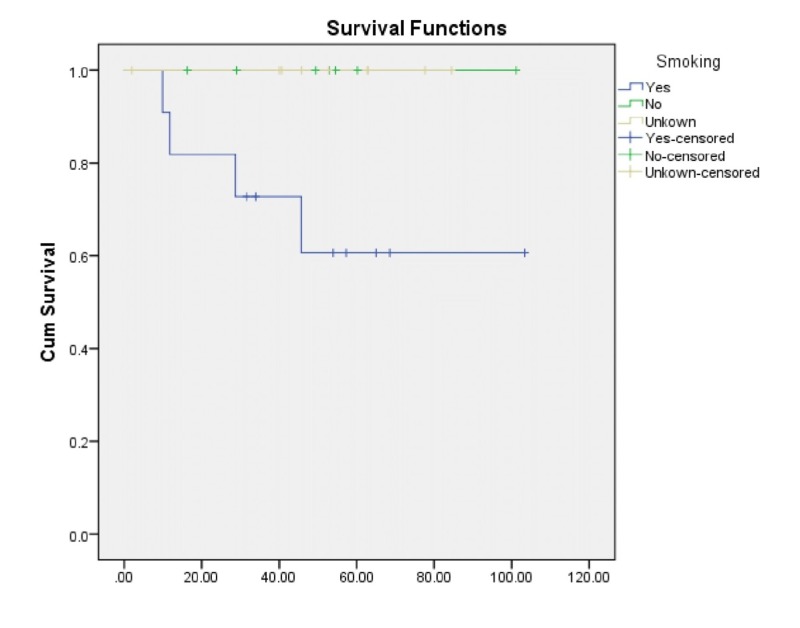
Local control rate based on smoking status

Lastly, we found that the patients who had an unknown or negative smoking history did well with a five-year LCR of 100%, while patients with a positive history of smoking had an LCR of 60.6% with a significant p-value (0.044) (Figure 3).

Disease-free survival (DFS)

**Figure 4:**
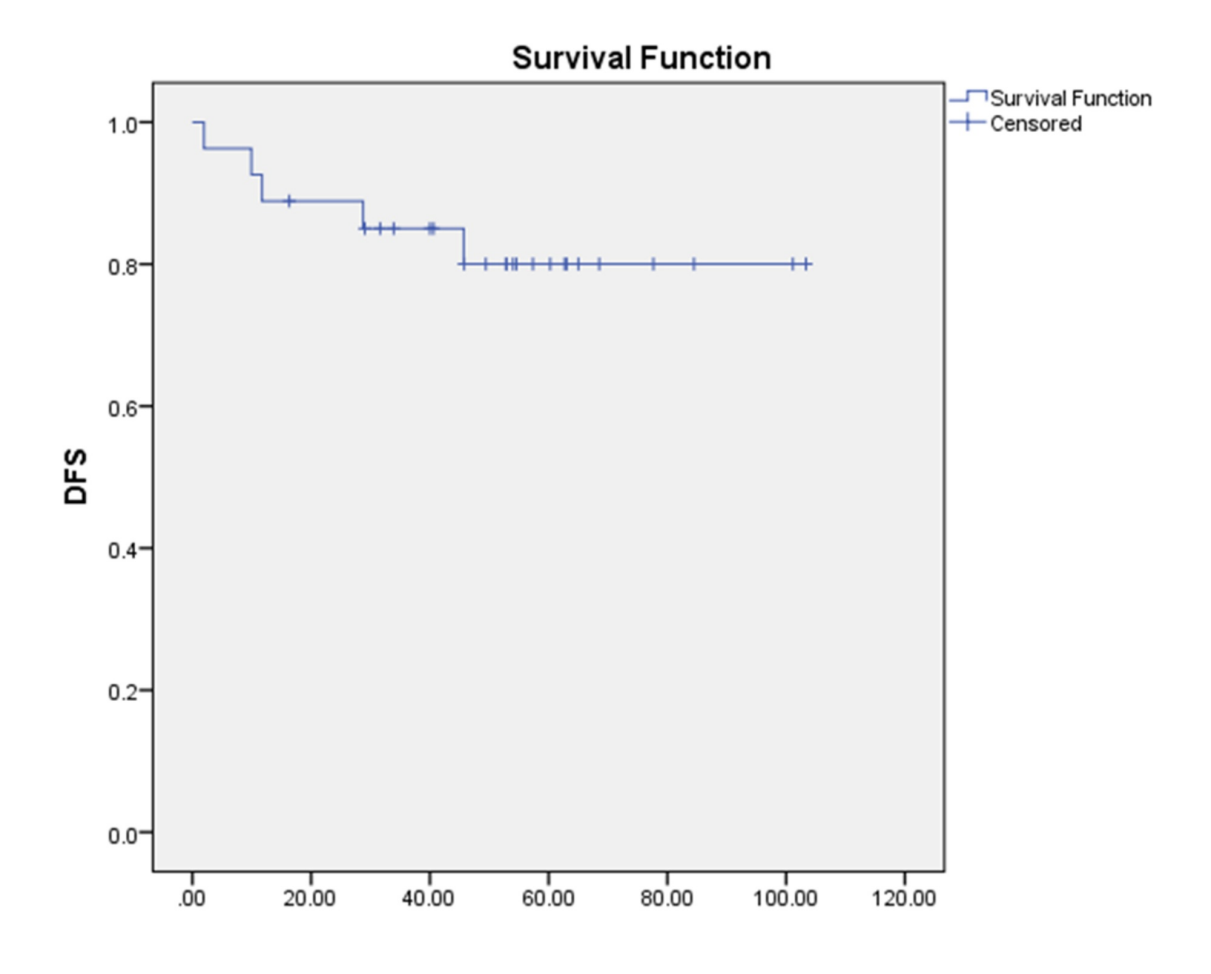
Disease-free survival (DFS)

During the follow-up, four patients developed local recurrences, one developed a second primary tumor (lung cancer) and received systemic chemotherapy. The five-year DFS of our cohort was 80.0% (Figure 4).

Death specific free survival (DSFS)

**Figure 5:**
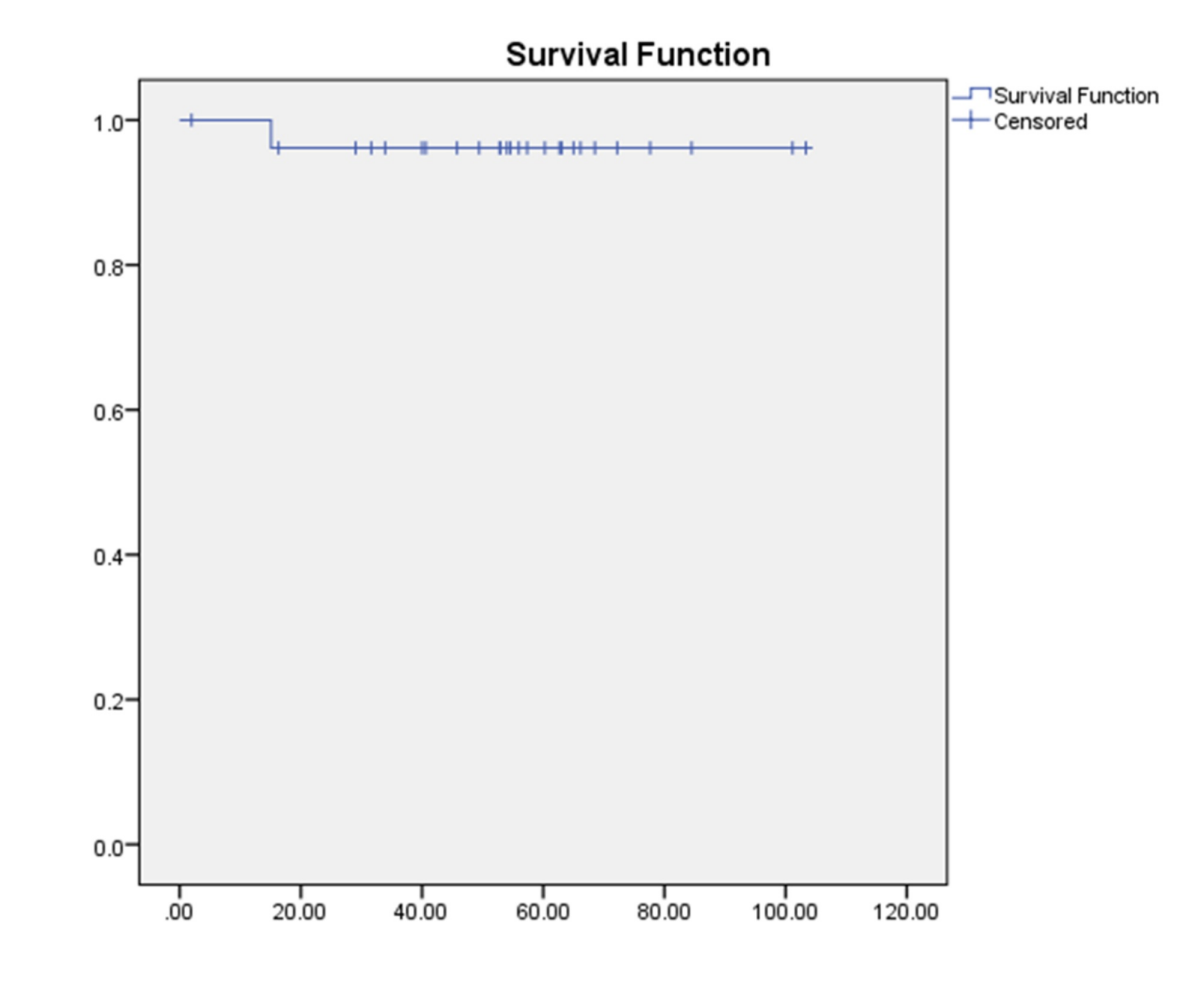
Death specific free survival (DSFS)

Our cohort showed only one patient who died due to progression of disease, laryngeal carcinoma, bringing the five-year DSFS to 96.2% (Figure 5).

Overall survival (OS)

**Figure 6:**
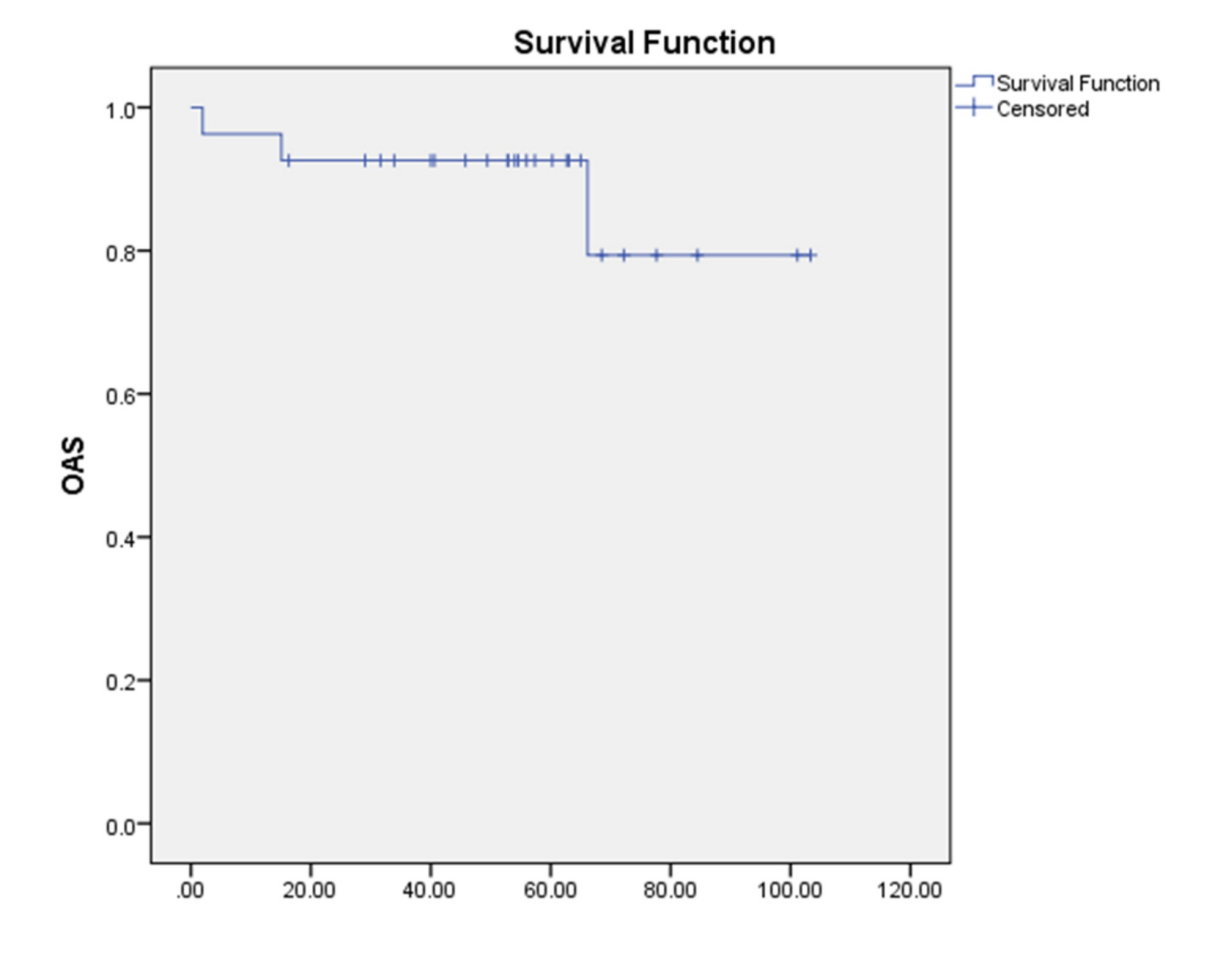
Overall survival OAS/OS = Overall survival

Among the 27 patients, three patients (11.1%) died during follow-up. The causes of death were progressive metachronous lung cancer (one patient), hepatocellular carcinoma (one patient), and progression of glottic carcinoma (one patient). The five-year OS rate of our cohort was 92.6% (Figure 6).

## Discussion

Radiotherapy is the treatment of choice with excellent laryngeal preservation rates in early glottic cancer. Various dose fractionation regimens have been used; however, trials incorporating hypofractionation schedules were found to be associated with significant local control rate (LCR) and quality of voice [6,7]. Our current retrospective study was aimed primarily to assess the efficacy of various fractionation schedules used in our institute for the treatment of EGC (T1, T2) in terms of local control rates.

LCR with a larynx preservation rate of 84.9% in our cohort was found in proximity to one large retrospective study conducted by Mendenhall et al. in which 519 patients with EGC showed a five-year LCR with a larynx preservation of 84.3% [6]. However, a study conducted at St James's Institute of Oncology at Leeds, UK showed a five-year LCR of 95.4% [12]. In our study, smoking history was found to be associated with poor LCR. Slightly inferior LCR in our research can be explained by (a) a predominantly male cohort (88.9%, n=24), as the male gender is known to be associated with decreased lower LCR after radiation therapy in previously published studies [6,15]; (b) four patients (14.8%) were treated with regular fractionation schedule, prolonged treatment time might have an impact on LCR [16]; (c) anterior commissure involvement in 11.1% (n=3) in our study, which has been addressed as an independent poor prognostic factor for local control; and (d) high percentage of positive smoking history (40.7%, n=11) [17-18].

The five-year OS rate was 92.1% in our cohort, which was found to be in agreement with other previously published studies [19-20]. No severe late toxicity was observed in our cohort, including in patients who underwent salvage laryngectomy, which was also found to be in agreement with published literature. An extensive study by Garden et al. reported 4% severe side effects following radiation therapy in 230 patients with T2 laryngeal carcinoma [21].

The limitations of our study included (a) a small sample population, (b) retrospective study design, (c) missing data regarding voice quality assessment, and (d) pretreatment hemoglobin was not evaluated.

## Conclusions

Radiation therapy for early glottic carcinomas in our local cohort of patients showed similar outcomes in terms of control rates and overall survival when compared to international data published in the literature. We were able to achieve a reasonable five-year local control rate with larynx preservation and excellent DFS, DSFS, and five-year OS rates. We found that only smoking had a significant correlation with LCR. However, large prospective trials are warranted to evaluate the efficacy of overall treatment time, dose per fraction of larger than 2 Gy, and smoking effect on the local control.
